# Fatty acid composition MRI of epicardial adipose tissue: Methods and detection of proinflammatory biomarkers in ST‐segment elevation myocardial infarction patients

**DOI:** 10.1002/mrm.30285

**Published:** 2024-09-25

**Authors:** John T. Echols, Shuo Wang, Amit R. Patel, Austin C. Hogwood, Antonio Abbate, Frederick H. Epstein

**Affiliations:** ^1^ Biomedical Engineering University of Virginia Charlottesville Virginia USA; ^2^ Division of Cardiovascular Medicine University of Virginia Charlottesville Virginia USA; ^3^ Robert M. Berne Cardiovascular Research Center University of Virginia Charlottesville Virginia USA; ^4^ Radiology University of Virginia Charlottesville Virginia USA

**Keywords:** cardiac MRI, epicardial adipose tissue, fatty acid composition

## Abstract

**Purpose:**

To develop a method for quantifying the fatty acid composition (FAC) of human epicardial adipose tissue (EAT) using accelerated MRI and identify its potential for detecting proinflammatory biomarkers in patients with ST‐segment elevation myocardial infarction (STEMI).

**Methods:**

A multi‐echo radial gradient‐echo sequence was developed for accelerated imaging during a breath hold using a locally low‐rank denoising technique to reconstruct undersampled images. FAC mapping was achieved by fitting the multi‐echo images to a multi‐resonance complex signal model based on triglyceride characterization. Validation of the method was assessed using a phantom comprised of multiple oils. In vivo imaging was performed in STEMI patients (*n* = 21; 14 males/seven females). FAC was quantified in EAT, subcutaneous AT, and abdominal visceral AT.

**Results:**

Phantom validation demonstrated strong correlations (*r* > 0.97) and statistical significance (*p* < 0.0001) between measured and reference proton density fat fraction and FAC values. In vivo imaging of STEMI patients revealed a distinct EAT FAC profile compared to subcutaneous AT and abdominal visceral AT. EAT FAC parameters had significant correlations with left ventricular (LV) end‐diastolic volume index (*p* < 0.05), LV end‐systolic volume index (*p* < 0.05), and LV mass index (*p* < 0.05).

**Conclusions:**

Accelerated MRI enabled accurate quantification of human EAT FAC. The relationships between the EAT FAC profile and LV structure and function in STEMI patients suggest the potential of EAT FAC MRI as a biomarker for adipose tissue quality and inflammatory status in cardiovascular disease.

## 1 INTRODUCTION

Epicardial adipose tissue (EAT), the adipose depot positioned between the myocardium and the visceral pericardium, has emerged as an important contributor to cardiovascular disease, particularly in obesity and metabolic heart disease.^1^, ^2^ In healthy individuals, EAT is a protective entity that provides local energy and thermal insulation to the myocardium. However, under pathological conditions that encourage excessive lipid accumulation, such as obesity and metabolic syndrome, EAT undergoes adverse changes involving adipocyte hypertrophy, alterations in its fatty acid composition (FAC), heightened macrophage infiltration,^3^, ^4^ and inflammatory cytokine secretion. These proinflammatory changes in EAT can have a significant influence on the pathophysiology of diverse forms of cardiovascular disease, including atrial fibrillation,^5^ coronary microvascular dysfunction,^6^ coronary artery disease,^7^ and heart failure.^7^, ^8^, ^9^, ^10^ Whereas similar changes may occur in other visceral adipose tissues, EAT is distinguished by its lack of defined boundaries with the heart. In particular, EAT shares an unobstructed microcirculation with the underlying myocardial tissue, which enables it to function as a local transducer of metabolic inflammation, fostering chronic low‐grade inflammation in the heart and contributing to disease development.^7^, ^8^

The FAC of the EAT, delineating the respective levels of saturated fatty acids (SFAs), monounsaturated fatty acids (MUFAs), and polyunsaturated fatty acids (PUFAs), has been proposed as a possible mediator of its proinflammatory properties.^11^ SFAs, notably, are linked to the activation of inflammatory pathways in adipose tissue. Specifically, SFAs activate toll‐like receptor 4, inducing nuclear factor kappa B translocation and resulting in the production of proinflammatory cytokines such as tumor necrosis factor alpha and interleukin‐6.^12^ These cytokines, in turn, can have detrimental effects on the adjacent myocardium, leading to cardiovascular dysfunction and adverse remodeling.^13^, ^14^ In contrast, certain MUFAs and PUFAs have been demonstrated to directly impede SFA‐induced inflammation through various pathways that inhibit nuclear factor kappa B activity.^15^, ^16^

Whereas previous research has explored MRI‐based metrics like EAT volume as clinically relevant descriptors of EAT,^17^, ^18^, ^19^ the characterization of FAC and proton density fat fraction (PDFF) with MRI introduces a novel dimension for assessment, potentially providing valuable measurements reflecting its proinflammatory state. Although FAC MRI techniques have been applied in the EAT of mice^20^ and abdominal,^21^ breast,^22^ and bone marrow^23^, ^24^ adipose tissues of humans, their application to human EAT remains unexplored.

To enable investigation of the role of FAC in human EAT and its potential as a biomarker for a proinflammatory state, we developed an accelerated MRI technique that can be applied during a breath hold. This noninvasive method enables the quantification of FAC within EAT and adjacent adipose depots, facilitating the assessment of levels of SFAs, MUFAs, and PUFAs. In this study, we present the detailed methodology for EAT FAC MRI and its application in identifying potential proinflammatory biomarkers in acute coronary syndrome patients following ST‐segment elevation myocardial infarction (STEMI). Whereas prior work has facilitated the measurement of EAT quantity, by integrating FAC mapping into the cardiac MRI exam we aim to develop potential new biomarkers to noninvasively assess EAT quality.

## 2 THEORY

### 2.1 Signal model

Triglycerides can be represented using a model that contains nine distinguishable hydrogen‐1 (^1^H) resonances m∈{A,…,I}.^25^ The complex exponential describing the phase of each resonance relative to water at a given time after excitation, t, is expressed as $\alpha_{m}\left(t\right)=e^{i\gamma B_{0}\left(\delta_{m}-\delta_{w}\right)t}$, where $B_{0}$ is the main magnetic field strength; $\delta_{m}$ and $\delta_{w}$ are the chemical shifts of triglyceride proton m and water proton w, respectively, in ppm; and γ is the gyromagnetic ratio. Chemical shifts for the different triglyceride resonances and their respective relative magnitudes are given in Table 1.

**TABLE 1:** Chemical shifts and relative magnitudes of triglyceride resonances.

Resonance m	Type	$\delta_{m}$ [ppm]	Relative Magnitude $\rho_{m}$
A	methyl	0.90	9
B	methylene	1.30	[(cl−4)×6]−(ndb×8)+(nmidb×2)
C	β‐carboxyl	1.60	6
D	α‐olefinic	2.02	(ndb−nmidb)×4
E	α‐carboxyl	2.24	6
F	diacyl	2.75	nmidb×2
G	glycerol	4.20	4
H	glycerol	5.19	1
I	olefinic	5.29	ndb×2

$\delta_{m}$, chemical shift; cl, chain length; ndb, number of double bonds; nmidb, number of methylene‐interrupted double bonds.

Consequently, the complex signal y of a voxel in a spoiled gradient echo (GRE) image with TE t can be decomposed into the contributions from water, W, and fat, F, and expressed as the nonlinear model:

(1) $$y\left(t\right)=\left(W+F\sum_{m}\rho_{m}\alpha_{m}\left(t\right)\right)e^{i2\pi \psi t}e^{\mathrm{i\phi}}e^{-R_{2}^{*}t}$$

where the off‐resonance frequency ψ is due to static field inhomogeneity, ϕ is the spatially dependent initial phase of the signal, $\rho_{m}$ is the relative magnitude of triglyceride resonance m, and $R_{2}^{*}$ is the transverse relaxation rate.

### 2.2 Triglyceride characterization

As shown by Hamilton et al., the relative sizes of the triglyceride peaks $\rho_{m}$ can be defined in terms of the number of double bonds (ndb), the number of methylene‐interrupted double bonds (nmidb), and the chain length (cl).^25^ Briefly, every triglyceride has nine terminal methyl protons (resonance A), five glycerol protons (four G and one H), and 12 protons from the carbonyl group (six E, six C). For every double bond, there are two olefinic (I) and four allylic (D) protons. Each methylene‐interrupted double bond coincides with two diallylic (F) and four olefinic (I) protons. The remaining number of methylene protons (B) is dependent on the chain lengths of the fatty acids. These relationships, shown in Table 1, can be incorporated into the signal model. Additionally, estimation of the chain length by either prior knowledge or use of the heuristic relationship cl=16.8+0.25ndb ^26^ further reduces the number of unknown parameters. This allows for the decomposition of the fat contribution to the signal model, F, into distinct subcomponents that are proportional to the characteristic triglyceride parameters. Thus, for any admixture of triglycerides:

(2) $$\begin{array}{c} F=F_{\mathrm{ntg}}+F_{\mathrm{ndb}}+F_{\text{nmidb}} \end{array}$$

where the real‐valued parameters $F_{\mathrm{ntg}}$, $F_{\mathrm{ndb}}$, and $F_{\text{nmidb}}$ are the fat subcomponents proportional to the number of triglycerides ntg, ndb, and nmidb, respectively. Using these subcomponents, we can calculate the absolute values of ndb and nmidb for the “mean triglyceride” within a voxel as:

(3) $$\begin{array}{c} \mathrm{ndb}=\frac{F_{\mathrm{ndb}}}{F_{\mathrm{ntg}}} \end{array}$$

(4) $$\begin{array}{c} \text{nmidb}=\frac{F_{\text{nmidb}}}{F_{\mathrm{ntg}}} \end{array}$$

Once ndb and nmidb have been determined, the fractions of unsaturated fatty acids (UFAs) as well as PUFAs can be calculated using:

(5) $$\begin{array}{c} \mathrm{UFA}=\frac{\mathrm{ndb}-\text{nmidb}}{3} \end{array}$$

(6) $$\begin{array}{c} \text{PUFA}=\frac{\text{nmidb}}{3} \end{array}$$

Subsequently, relative values for the SFAs and MUFAs can be determined such that UFA+SFA=100% and UFA=MUFA+PUFA. ^21^

### 2.3 Least‐squares variable projection

For a multi‐echo GRE acquisition with N TEs $\left[t_{1},\ldots ,t_{N}\right]$, the acquired MR signal as a complex column vector $y={\left[y\left(t_{1}\right),\ldots ,y\left(t_{N}\right)\right]}^{T}$ can be expressed in matrix form in terms of the real‐valued, linear parameter vector $x=\left[W,F_{\mathrm{ntg}},F_{\mathrm{ndb}},F_{\text{nmidb}}\right]$ as:

(7) $$\begin{array}{c} \mathrm{\Psi Α}xe^{\mathrm{i\phi}}=y \end{array}$$

where

$${Α}_{N\times 4}=\left[\begin{array}{l} \alpha_{1w}\\ \vdots \\ \alpha_{\mathrm{Nw}} \end{array}\begin{array}{lll} \alpha_{1A} & \cdots & \alpha_{1I}\\ \vdots & \ddots & \vdots \\ \alpha_{\mathrm{NA}} & \cdots & \alpha_{\mathrm{NI}} \end{array}\right]\underset{\rho }{\underbrace{\left[\begin{array}{llll} 1 & 0 & 0 & 0\\ 0 & 9 & 0 & 0\\ 0 & 6\left(\mathrm{cl}-4\right) & -8 & 2\\ 0 & 6 & 0 & 0\\ 0 & 0 & 4 & -4\\ 0 & 6 & 0 & 0\\ 0 & 0 & 0 & 2\\ 0 & 2 & 0 & 0\\ 0 & 2 & 0 & 0\\ 0 & 1 & 0 & 0\\ 0 & 0 & 2 & 0 \end{array}\right]}}$$

and $\Psi_{N\times N}=\mathrm{diag}\left[e^{i2\pi \psi t_{n}}\right]\;\left(n=1,\ldots ,N\right)$. The ρ matrix describes the relative weights of each resonance for each water and fat signal component, and the terms $\alpha_{\mathrm{nw}}=e^{-R_{2}^{*}t_{n}}$ and $\alpha_{\mathrm{nm}}=e^{i\gamma B_{0}\left(\delta_{m}-\delta_{w}\right)t_{n}}e^{-R_{2}^{*}t_{n}}$ account for transverse relaxation effects and chemical shift–induced frequency changes of the water and fat protons.

The objective of the proposed fatty acid quantification method is to determine the unknown parameter vector x and the confounding parameters ψ, $R_{2}^{*}$, and ϕ. When the confounding parameters are known and Ψ and Α are given, then a real‐valued estimate of x can be determined in the least‐squares sense as:

(8) x^=ReΑHA−1ReΑHΨHye−iϕ

The confounding parameters can be found by minimizing the squared error residual function:

(9) JR2*,ψ,ϕ=y−ΨAReΑHA−1ReΑHΨHye−iϕeiϕ2

We can analytically derive the initial phase term that minimizes Equation (9), $\hat{\phi }$, for a given ψ and $R_{2}^{*}$ as:

(10) ϕ^=12argΑHΨHyTReΑHA−1ΑHΨHy

Inserting Equation (10) into Equation (9) results in a nonlinear functional that depends solely on ψ and $R_{2}^{*}$ and can be used to determine the remaining confounding parameters.

### 2.4 Locally low‐rank property of chemical shift imaging

Signal models based on chemical shift encoding, such as the one described in this work, yield images at multiple TEs that are superpositions of a limited number of spectral components. Using the common assumption that the phase variation is locally smooth,^27^, ^28^ for small local regions of the image the phase variation from field inhomogeneities can be considered constant. Thus, the signal evolution for a sufficiently small image patch can be considered to be spectrally sparse, containing only contributions from the modeled water and fat components.

In order to exploit the spectral sparsity, small local image regions with P elements at all N TEs can be correlated in a Casorati matrix $C_{P\times N}$. In the context of the previously described signal model, the rank of this matrix is at most four and is in general restricted by the number of modeled spectral components such that $\mathrm{rank}\left(C_{P\times N}\right)\leq \dim\left(x\right)$. This locally low‐rank (LLR) property means that at most the first four singular values and their respective singular vectors contain significant information about the dominating components, whereas the remaining data consists primarily of noise.^29^

The LLR characteristic of multi‐echo imaging can be leveraged to accelerate multi‐echo GRE imaging by performing nuclear norm minimization of undersampled images.^30^ This can be done through singular value hard thresholding (SVHT) of the Casorati matrix using a complex‐valued singular value decomposition.^30^ In SVHT, each singular value $\sigma_{n}$ of the Casorati matrix that lies below a chosen threshold λ is set to 0. As described by Gavish and Donoho, the optimal threshold for a square matrix (i.e., a Casorati matrix when P=N) can be selected using the formula^31^:

(11) $$\begin{array}{c} \lambda =\frac{4}{\sqrt{3}}*\sqrt{N}\sigma_{\mathrm{opt}} \end{array}$$

Here, $\sigma_{\mathrm{opt}}$ represents the known white noise level observed in the matrix. Because the rank of $C_{P\times N}$ is constrained by the number of modeled spectral components, the white noise level can be approximated as $\sigma_{\mathrm{opt}}\approx \sigma_{\dim\left(x\right)+1}$.

## 3 METHODS

### 3.1 Pulse sequence design

**FIGURE 1:**
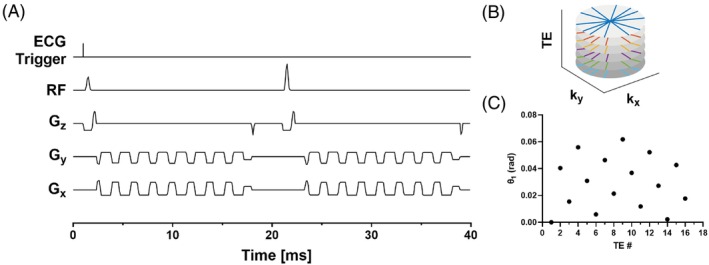
(A) Pulse sequence diagram depicting the acquisition of one radial spoke for 16 TEs. (B) Example radial trajectories for the first six spokes and five TEs. (C) Projection angles for the first spoke for each TE.

An electrocardiogram (ECG)‐gated multi‐echo radial GRE sequence with monopolar readout gradients was developed using flyback gradient pulses to obtain 16 images at different TEs. To achieve a shorter echo spacing, the acquisitions at the 16 TEs were distributed across two RF excitations. Each excitation consisted of an echo train with eight echoes, starting at TEs of 1.8 and 2.7 ms, respectively. This approach yielded a sequence of 16 GRE images at evenly spaced TEs from 1.8 to 15.3 ms with an effective echo spacing of 0.9 ms. A sequence diagram for an example radial trajectory is shown in Figure 1A. This choice of TEs and echo spacing was selected to reduce the variance in the estimates of $W,F_{f},F_{\mathrm{ndb}},F_{\text{nmidb}}$ in accordance with the recommendations outlined by Berglund et al.^32^

Following each ECG trigger, eight excitations were performed with a TR of 18.5 ms between excitations. Variable flip angles of 22°, 24°, 26°, 29°, 32°, 38°, 48°, and 86° were used for the successive excitations, which were calculated to maintain an approximately constant signal level for the chosen TR and an assumed T_1_ for adipose tissue of 350 ms. The use of these flip angles reduces the impact of T_1_ relaxation on signal changes while maximizing the overall signal generated.

The acquisition provides a total of 48 radial spokes at each TE over 12 heartbeats during a single breath‐hold and results in approximately rate‐6 accelerated images for a matrix size of 192 × 192. To ensure different aliasing patterns for each GRE image, a golden angle rotated stack‐of‐stars sampling strategy was used. Example trajectories for six spokes and five TEs are shown in Figure 1B, and angles for the first spoke of 16 TEs are shown in Figure 1C. Generally, for $N_{r}$ total projections the projection angle θ for the $i^{\mathrm{th}}$ projection and $n^{\mathrm{th}}$ TE is calculated as:

(12) $$\begin{array}{ll} \theta \left(i,n\right)= & \;\mathrm{mod}\left(\left(i-1\right)\times \pi \times \frac{\sqrt{5}-1}{2},\pi \right)\\ & +\mathrm{mod}\left(\left(n-1\right)\times \frac{\pi }{N_{r}}\times \frac{\sqrt{5}-1}{2},\frac{\pi }{N_{r}}\right) \end{array}$$

### 3.2 Image reconstruction and FAC mapping

**FIGURE 2:**
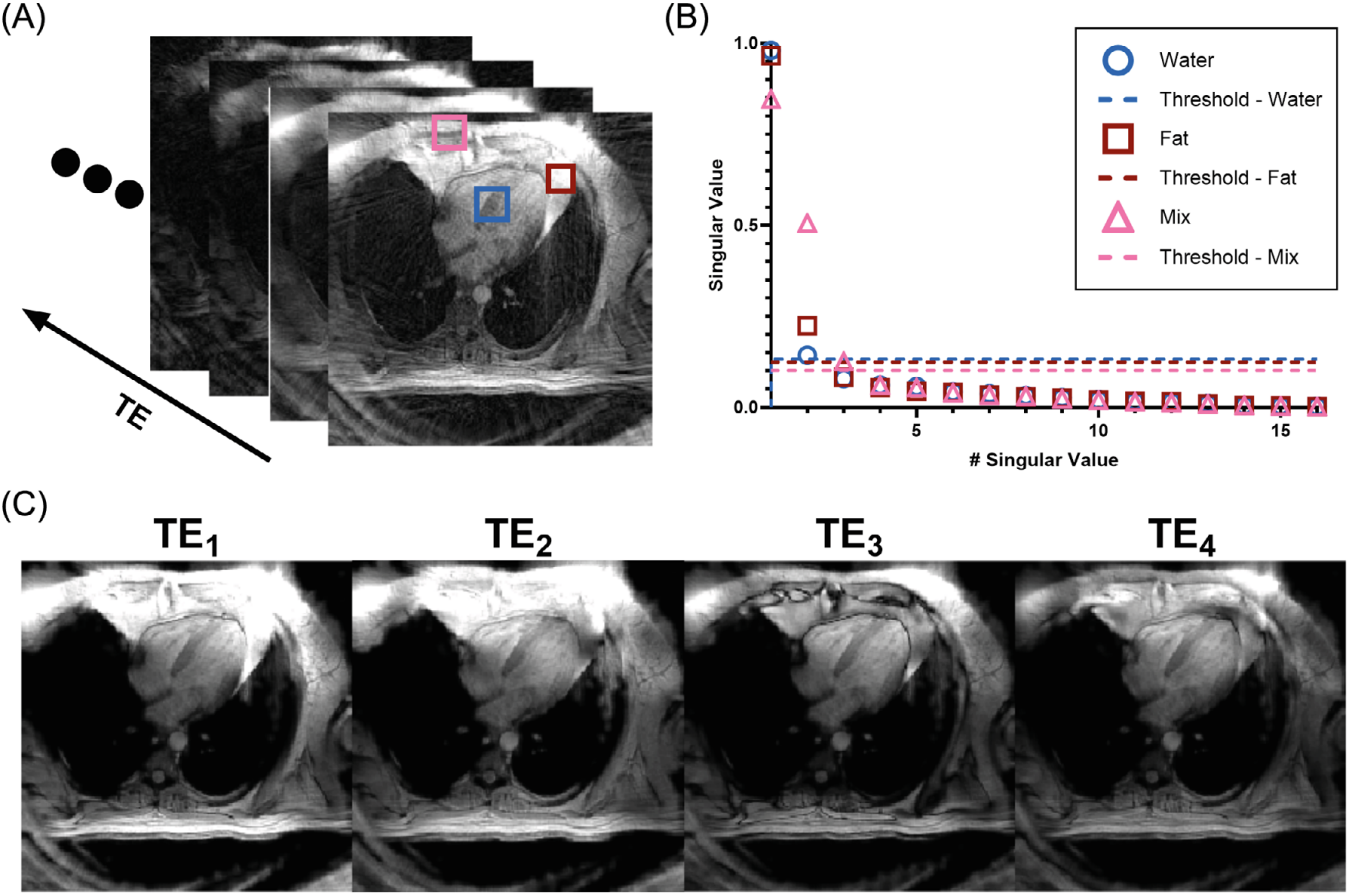
(A) Undersampled multi‐echo gradient echo images with patches drawn over regions of varying water and fat content. (B) The singular values and cutoff thresholds calculated for each patch. (C) GRE images at the first four TEs after performing singular value thresholding showing reduction of streaking artifacts. GRE, gradient echo.

Prior to image reconstruction, the k‐space trajectories for each radial spoke were calculated from the gradient waveforms. Subsequently, spoke trajectories were corrected to account for gradient delays along each spatial axis, which were determined from independently acquired calibration data.^33^, ^34^ The gradient delays were determined to be 1.7, 1.5, and 1.7 μs for the sagittal, coronal, and transverse axes, respectively. Coil compression of the multi‐coil, multi‐echo k‐space data was performed at a tolerance of 5% of the total variance to generate multi‐echo k‐space data for a reduced number of virtual coils.^35^ The virtual coil images at each TE were then reconstructed using the nonuniform fast Fourier transform.^36^ The ESPIRiT^37^ method was used to calculate the virtual coil sensitivity maps using the virtual coil images from the first TE. The resulting sensitivity maps were then applied to combine the virtual coil images for each TE, generating images that contain undersampling artifacts. As the sequence employed golden angle radial sampling, undersampling artifacts appear similar to random noise that is different for each TE. To denoise these images, an LLR constraint was applied to image patches through SVHT. Because this LLR reconstruction approach utilizes an adaptive thresholding method, the only parameter that must be chosen isthe patch size, which was set to 4 × 4. The singular value distribution of 4 × 4 image patches and their respective thresholds are shown in Figure 2, along with example images after LLR denoising.

Following LLR denoising, field map ψ and extrinsic transverse relaxation $R_{2}^{*}$ estimates were generated. As shown by Berglund et al., the residual function (Equation (9)) has multiple local minima along the ψ dimension, and the correct minimum can be found by imposing spatial smoothness of the off‐resonance map using a discrete whole‐image optimization scheme.^28^ This approach was employed for the minimization of $R_{2}^{*}$ and ψ using $R_{2}^{*}$ values discretized between 0 and 300 s^−1^ with increments of 2 s^−1^. Subsequently, Equation (10) is then used to calculate *ϕ*.

For each voxel, the parameter vector x is derived by inserting the confounding parameters ψ, $R_{2}^{*}$, and *ϕ*, as well as the noise‐filtered complex echo images, into Equation (8). An initial estimation of x is first made for each voxel using a simplified signal model that incorporates the $F_{\text{nmidb}}$ term within the $F_{\mathrm{ndb}}$ term using an assumed ratio of nmidb to ndb. The final solution for x is subsequently found by restricting all terms in x to be between 0.5 and two times their initial estimates, using the same assumed ratio of nmidb to ndb to calculate an initial estimate of $F_{\text{nmidb}}$ from the initial estimate of $F_{\mathrm{ndb}}$. The bounded least squares problem is then solved using the trust region interior‐point algorithm.^38^ After x has been determined, ndb and nmidb (using absolute elements of x) and UFA and PUFA (Equations (5) and (6)) are calculated and then subsequently used to calculate SFA and MUFA. The PDFF is also calculated as F/(F+W), which describes the portion of the total signal that is produced by fat protons. The described framework was implemented in MATLAB r2022b (MathWorks, Natick, MA).

### 3.3 NMR spectroscopy

As a reference method, NMR spectroscopy was used to characterize the *ndb* and *nmidb* and SFA, MUFA, and PUFA fractions of various oils. Samples were placed in NMR tubes with width of 5 mm and length of 7 in. A standard 1D ^1^H pulse sequence was used on a Bruker AVIII‐600 spectrometer to acquire 1D ^1^H spectra. A total of eight scans were performed using acquisition parameters that included a spectral width of 6.6 kHz and a relaxation delay of 7.52 s. Spectral analysis was performed in Mnova v14.2.1 (Mestrelab Research SL, Santiago de Compostela, Spain) to calculate the areas of the peaks located at the chemical shifts described in Table 1. During processing, a Gaussian window function with a line broadening factor of 0.1 Hz was applied; manual phase correction was performed; and polynomial baseline correction was used. Estimates of the *cl*, *ndb*, and *nmidb* were subsequently determined through a least‐squares minimization of the calculated areas of each peak to the relative areas described in Table 1; and SFA, PUFA, and MUFA fractions were quantified from the resulting estimates using Equations (5) and (6).

### 3.4 Oil phantom

**FIGURE 3:**
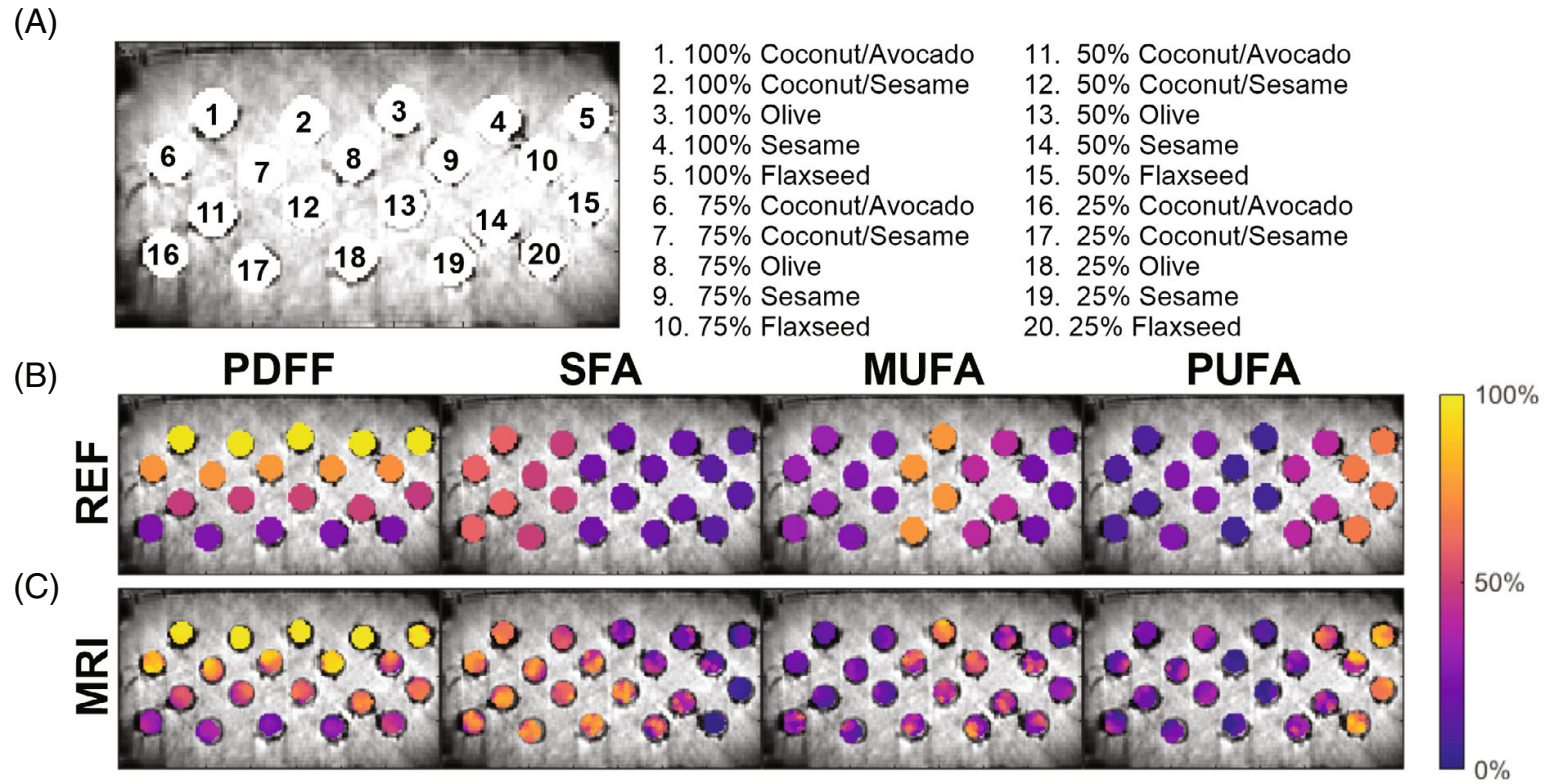
(A) Oil emulsion phantom layout; (B) synthetic parameter maps depicting the FAC reference values; and (C) MRI‐measured FAC maps of the oil emulsion phantom used for validation. SFA, MUFA, PUFA, and PDFF maps are plotted as colormaps over the combined water and fat magnitude image. FAC, fatty acid composition; MUFA, monounsaturated fatty acid; PDFF, proton density fat fraction; PUFA, polyunsaturated fatty acid; SFA, saturated fatty acid.

A phantom was constructed from commercially available oils (coconut, olive, avocado, sesame, flaxseed). Five oil samples were made from olive, sesame, and flaxseed oils as well as a 50/50 mixture of coconut/avocado oil and a 25/75 mixture of coconut/sesame oil. Oil‐in‐water emulsions at concentrations of 25%, 50%, and 75% were made based on the method described by Bush et al. for each oil sample.^39^ The various emulsions and 100% oil samples were placed into 50 mL polypropylene vials, which were then placed into a water bath inside of a rectangular polypropylene container. An illustration of the phantom layout is depicted in Figure 3A. The chosen oils contain primarily chain lengths found in human adipose tissue and span a wide range of FACs that encompass the expected FAC range of human adipose tissue.^40^

### 3.5 Phantom imaging and validation

Phantom imaging was performed using a 3 T MRI scanner (Magnetom Prisma, Siemens Healthcare, Erlangen,Germany) using the previously described pulse sequence and reconstruction protocol. The NMR measured chain lengths and nmidb to ndb ratios for each oil were used as needed to generate the matrix of relative proton amplitudes ρ. The FOV was 450 × 450 mm and slice thickness was 8 mm. The volumetric oil to water ratios for each emulsion were converted into nominal PDFF values using their respective densities, molecular masses, and number of protons per molecule. A region of interest (ROI) was drawn for each oil sample. To assess PDFF accuracy in water only voxels, additional ROIs were drawn at five locations within the water bath adjacent to the 100% oil samples. For assessment of FAC, the calculation of the PUFA fraction (Equation (6)) assumes that fatty acid chains contain either zero, one, or two double bonds, which is accurate for roughly 98% of fatty acids found in human adipose tissue. However, some plant oils, such as flaxseed oil, contain significant amounts of triunsaturated fatty acids. Thus, for all oils, the calculation of measured PUFA fractions was adapted using $\text{PUFA}=\frac{\text{nmidb}}{3}-F_{\text{trifa}}$, where $F_{\text{trifa}}$ denotes the fraction of triunsaturated fatty acids. The fixed $F_{\text{trifa}}$ value was estimated for each respective oil using their NMR‐measured FACs.

The difference between the average FAC MRI values and the reference values for each oil was calculated for each sample. Agreement of PDFF and FAC values between the two methods was assessed using linear regression. Pearson's linear correlation coefficient r and its 95% confidence intervals were used as a measure for linearity and *p*‐values for testing the null‐hypothesis that there is no linear relationship between the measurements and reference values were calculated. For FAC values, only samples with a reference PDFF of 100% were used for linear analysis because these samples are the closest to human adipose tissue in terms of PDFF.

### 3.6 In vivo imaging protocol and image analysis

In vivo imaging was performed using a 3 T MRI scanner (Magnetom Skyra) with chest and spine phased‐array receiver coils (20 to 34 channels). Shimming was performed using a localized protocol with the shim volume placed over the heart. FAC images were acquired at end diastole for a four‐chamber long‐axis slice and for basal, mid‐, and apical short‐axis slices using a FOV of 400 × 400 mm and a slice thickness of 8 mm. A chain length of 17.4 and a nmidb to ndb ratio of 0.26 was used as needed to generate the matrix of relative proton amplitudes ρ based on literature reported FACs of human adipose tissue.^4^, ^40^ Images and parameter maps were reconstructed using the processes described above. The EAT, subcutaneous adipose tissue (SAT), and abdominal visceral adipose tissue (VAT) depots were manually segmented using the images of total fat content F. ROIs for each tissue included voxels from artifact free regions with a PDFF >75% and excluded voxels along the border of the segmentation to limit the potential inclusion of partially volumed voxels. The mean PDFF and FAC values for each depot of each subject were calculated from all slices with an ROI of at least 50 pixels and used as the comparative metric for each subject.

### 3.7 In vivo reproducibility assessment

To determine the reproducibility of the proposed method, a scan–rescan study in volunteers was performed. Five subjects (three males/two females; age 35.6 ± 10.6; body mass index 30.6 ± 1.8) completed a cardiac MRI protocol that included EAT FAC imaging. All subjects signed an informed consent form in accordance with an institutional review board–approved protocol. Each scan in the protocol was performed three times, with the first scan serving as the baseline reference scan, a second scan immediately following the first without changes to the slice or subject position, and a third scan after removing the participant from the scanner and repositioning the subject. To assess agreement between measurements, Bland–Altman analysis was performed for EAT and SAT using the first scan as the reference.

### 3.8 Application to STEMI patients

Twenty‐one patients with STEMI underwent cardiac MRI, including EAT FAC imaging, within 96 h of reperfusion. This patient group was selected for an initial evaluation of EAT FAC MRI because these patients have a substantial volume of EAT, whereas healthy volunteers often have very small EAT volumes, and the volume of EAT in STEMI patients has been shown to be associated with severity of STEMI.^41^ All patients signed an informed consent form (HSR220072). The patient demographics were as follows: 14 (66%) were male and seven (33%) were female, with a mean ± SD age of 62.6 ± 10.4 years and a mean body mass index of 29.2 ± 5.6 kg/m^2^. In addition to EAT FAC MRI, patients also underwent cine imaging to assess left ventricular (LV) morphology and late gadolinium‐enhanced imaging to assess infarct size using the protocol outlined by the Society for Cardiovascular Magnetic Resonance.^42^ LV end diastolic volume (EDV), end systolic volume (ESV), and mass were calculated from a short‐axis stack of cine images and indexed by height. EAT volume was additionally calculated from the short axis cine images using the method described by Nelson et. al.^43^ Infarct size was calculated as a percentage of the LV mass using the short‐axis late gadolinium‐enhanced imaging images.

Comparisons were performed between the mean PDFF, SFA, MUFA, and PUFA values for each depot across all patients using a Welch and Brown–Forsythe analysis of variance. Associations between EAT FAC, infarct size, LV function, and LV structure were assessed using Pearson's linear correlation coefficient r and associated *p*‐values. A value of *p* < 0.05 was considered statistically significant. All statistical calculations were performed using GraphPad Prism version 10.0.2 for Windows (GraphPad Software, Boston, MA).

## 4 RESULTS

### 4.1 Phantom validation

**FIGURE 4:**
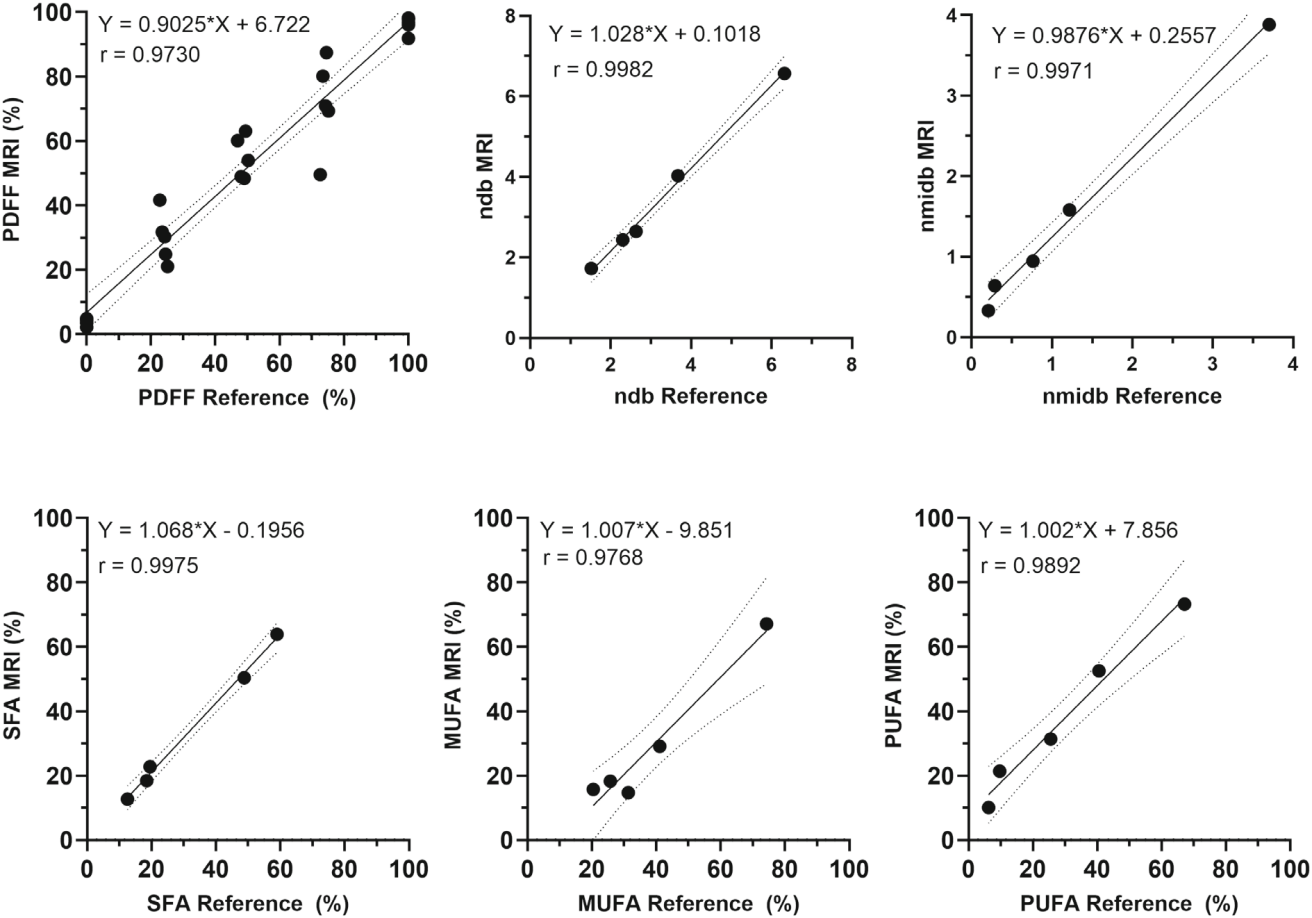
Linear regression plots between the mean MRI‐measured PDFF and FAC of the various oil emulsions and respective reference values. The dashed lines indicate 95% confidence intervals. For *ndb*, *nmidb*, SFA, MUFA, and PUFA values, only samples with a reference PDFF of 100% were used for linear analysis because they are the closest to human adipose tissue in terms of PDFF.

Results from the phantom validation demonstrated strong correlations (*r* > 0.97) and statistical significance (*p* < 0.0001) between the reference and measured PDFF and FAC values, showing the accuracy of the FAC MRI technique. Figure 3B depicts synthetic PDFF and FAC maps generated using the NMR measured reference values, and Figure 3C shows the MRI FAC maps. Regression analysis (Figure 4) demonstrated a slope of 0.90 with a bias of 6.72 for PDFF and produced slopes of 0.98 and 1.03 with biases of 0.10 and 0.26 for *ndb* and *nmidb*, respectively. Calculations of SFA, MUFA, and PUFA showed slopes ranging 1.00 to 1.07 and biases ranging from −9.85 to 7.86. The mean absolute error in PDFF percentage across all samples was 6.42. Mean absolute errors in *ndb*/*nmidb*/SFA/MUFA/PUFA were 0.55/0.23/12.38/13.37/7.69 across all samples, 0.31/0.24/1.99/9.57/7.91 for 100% oil emulsions, 0.59/0.30/12.04/13.97/10.08 for 75% oil emulsions, 0.53/0.17/16.74/15.68/5.80 for 50% oil emulsions, and 0.77/0.21/18.75/14.25/6.96 for 25% oil emulsions. This demonstrates good performance across oil concentrations with the best performance at the higher oil concentrations that are typical of human adipose tissue.

### 4.2 In vivo reproducibility

**FIGURE 5:**
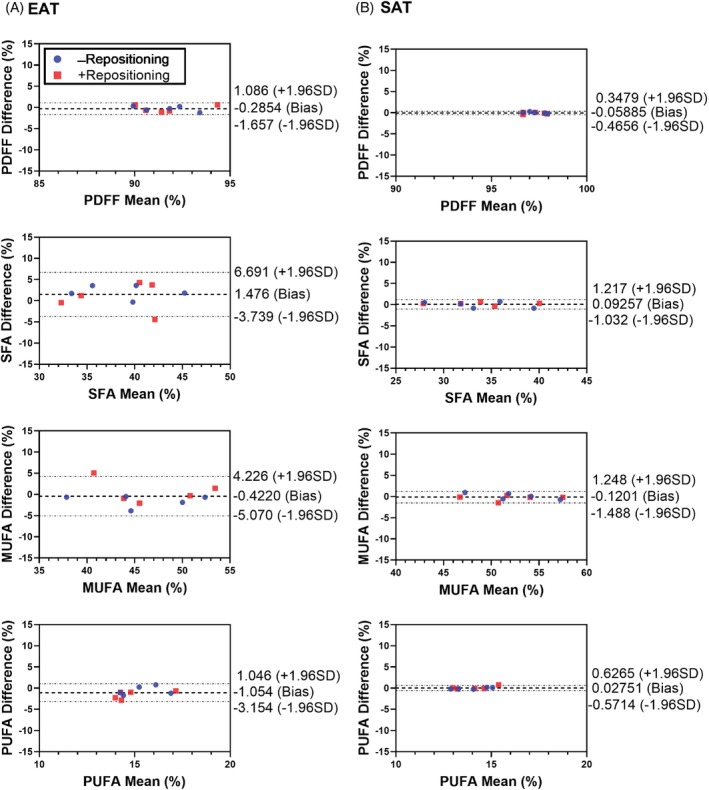
Bland–Altman analyses demonstrating the reproducibility and repeatability of measurements in both the EAT and SAT. Analyses include differences between the initial scans of subjects and subsequent scans without repositioning the subject (blue) and with repositioning the subject (red). EAT, epicardial adipose tissue; SAT, subcutaneous AT.

The results of the reproducibility study are depicted in Bland–Altman plots shown in Figure 5. Biases for PDFF and FAC measurements in the EAT and SAT, respectively, ranged from −1.05% to 1.48% and −0.12% to 0.09%, with confidence intervals ranging from −5.07% to 6.69% and −1.48% to 1.25%. The respective mean absolute differences in PDFF/SFA/MUFA/PUFA were 0.65%/2.51%/1.73%/1.27% in the EAT and 0.17%/0.49%/0.51%/0.21% in the SAT.

### 4.3 FAC mapping in STEMI patients

**FIGURE 6:**
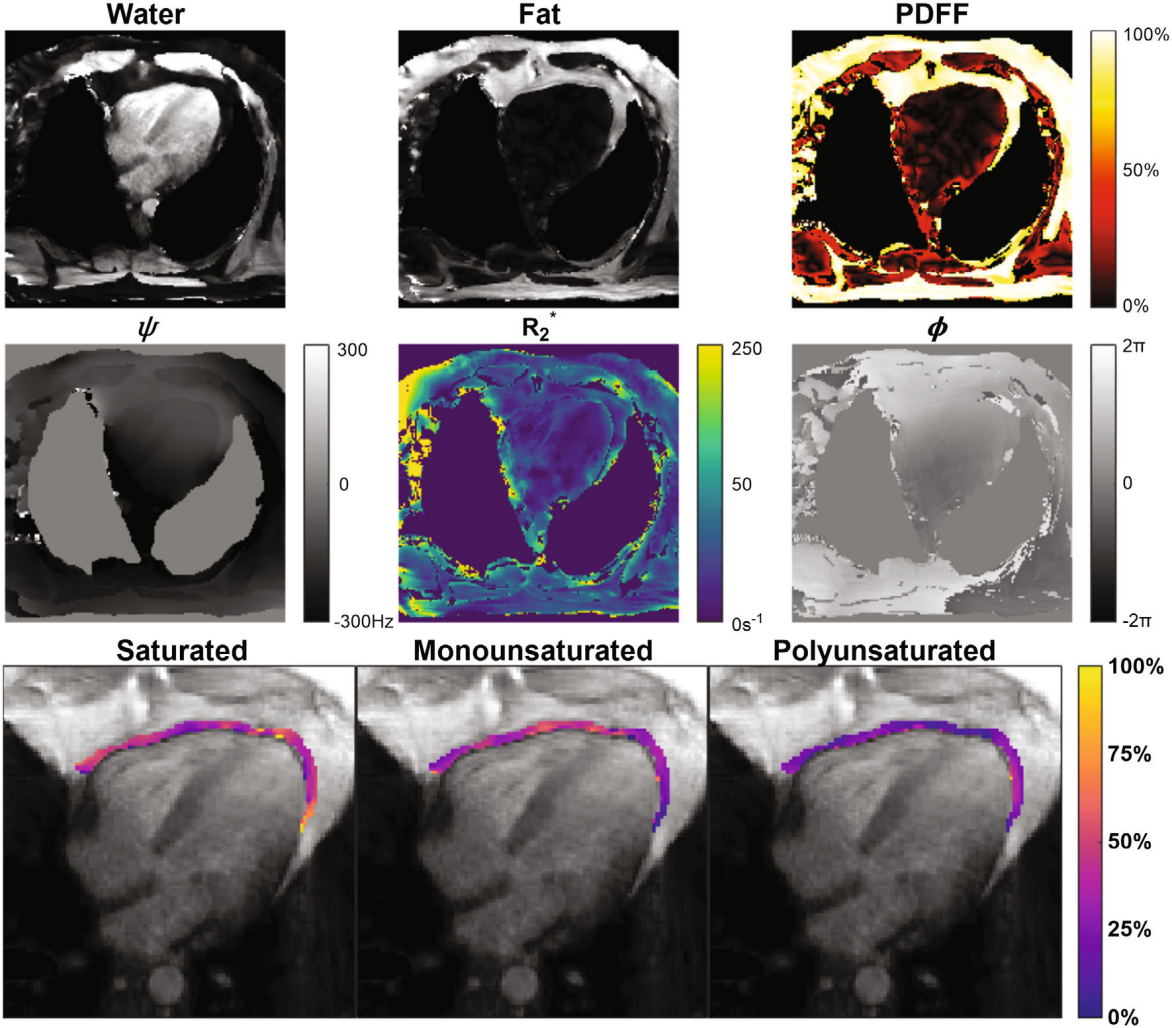
Images of a four‐chamber slice taken from a representative STEMI patient. Shown are images of the water component W; total fat component F; and maps of PDFF, $\psi ,R_{2}^{*}$, and ϕ. EAT FAC maps of SFA, MUFA, and PUFA are displayed. The EAT region was manually contoured, and the GRE magnitude image at the first TE is used as the background. STEMI, ST‐segment elevation myocardial infarction.

**FIGURE 7:**
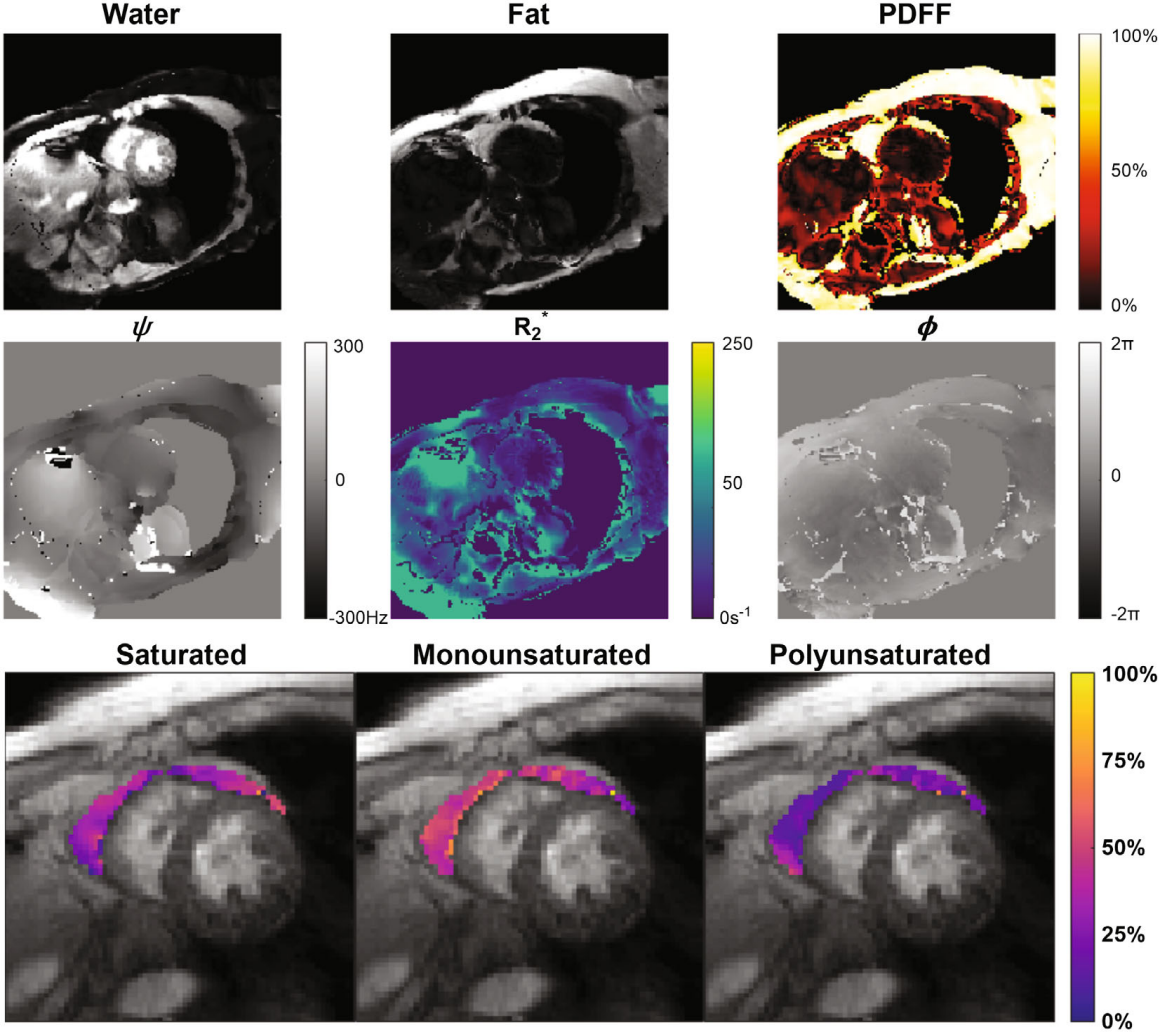
Images of a midventricular short‐axis slice taken from a representative STEMI patient. Shown are images of the water component W and total fat component F, as well as maps of PDFF, $\psi ,R_{2}^{*}$, and ϕ. EAT FAC maps of the SFA, MUFA, and PUFA are displayed. The EAT region was manually contoured, and the GRE magnitude image at the first TE is used as the background.

**FIGURE 8:**
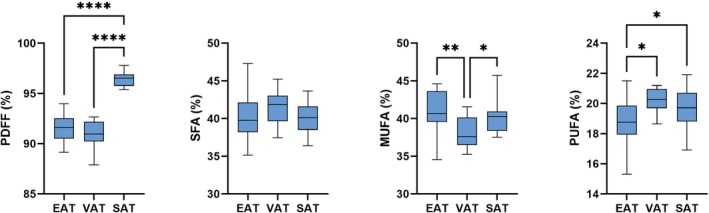
FAC and PDFF values for EAT, VAT, and SAT in STEMI patients (*N* = 21). Box plots depict means, minimums, maximums, and quartiles of each group. *p < 0.05; **p < 0.01; ****p < 0.0001.

Example water and fat images, as well as maps of PDFF, ψ, $R_{2}^{*}$, and ϕ, along with EAT FAC maps of SFA, MUFA, and PUFA fractions from representative STEMI patients are shown for a long‐axis and mid‐ventricular short‐axis slice in Figures 6 and 7, respectively. In slices containing at least one pixel of the respective fat depot, the average (± SD) ROI size per slice was 189 ± 57 pixels for the EAT, 1430 ± 952 pixels for the SAT, and 1165 ± 616 pixels for the VAT. The average number of total ROI pixels from all slices for each patient was 533 ± 285 for the EAT, 4493 ± 3152 for the SAT, and 2688 ± 1636 for the VAT. For all patients, PDFF, SFA, MUFA, and PUFA results for each adipose depot are summarized in Figure 8. PDFF was significantly lower in the EAT (*p* < 0.0001) and VAT (*p* < 0.0001) compared to the SAT. Compared to the VAT, MUFA levels were significant elevated in the EAT (*p* = 0.005) and SAT (*p* = 0.03). The EAT also had significantly lower levels of PUFAs than the VAT (*p* = 0.01) and SAT (*p* = 0.04). In this way, for STEMI patients, the FAC profile of EAT was distinctly different than that of SAT and VAT.

**FIGURE 9:**
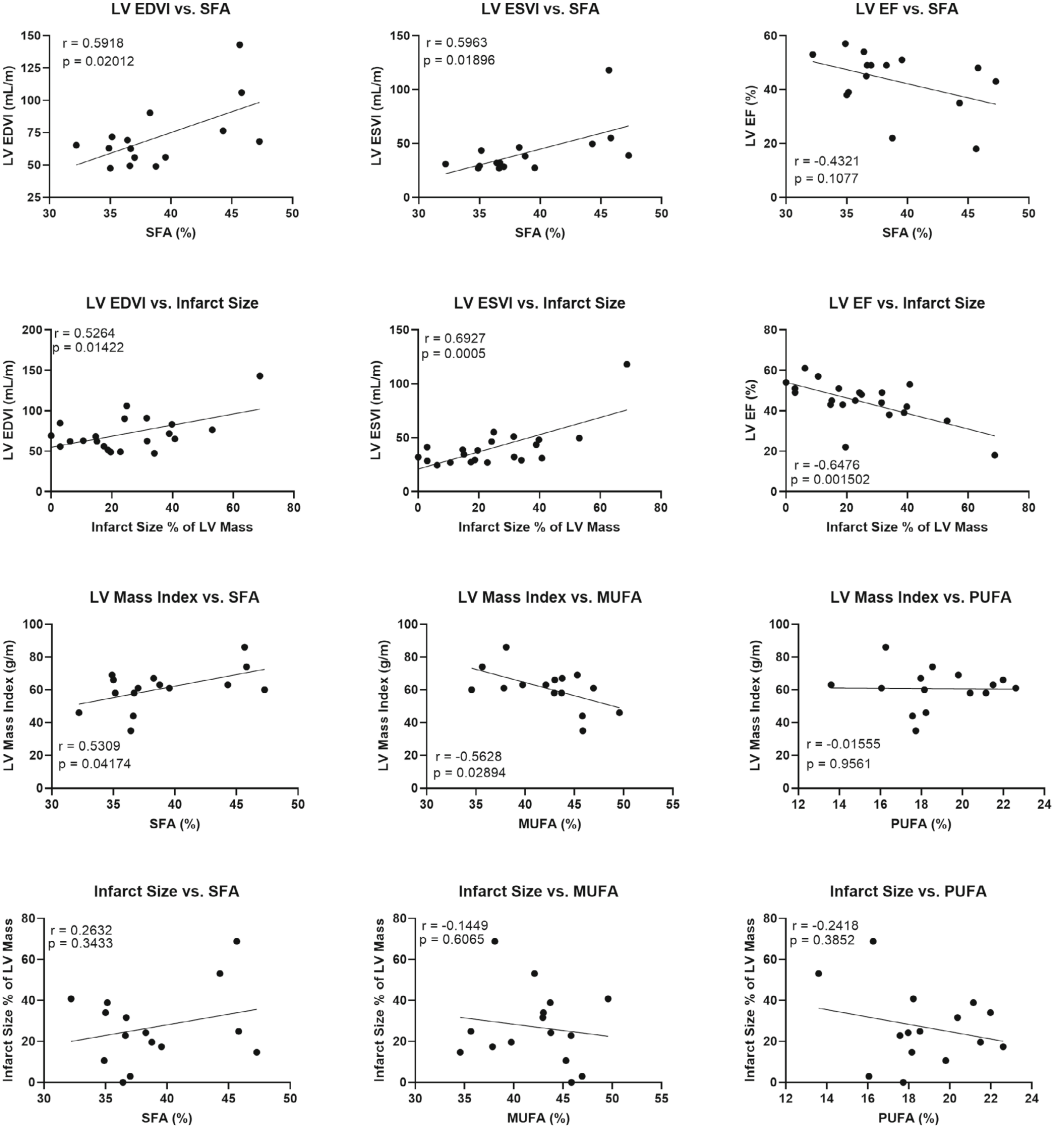
Scatter plots showing various linear relationships between EAT FAC, infarct size, and LV structure and function. Pearson's r and associated *p*‐value are listed for each plot. LV, left ventricular.

Correlations between EAT FAC and measurements of LV structure and function revealed several relationships, as shown in Figure 9. LV EDVI and LV ESVI were positively correlated with EAT SFA (*p* ≤ 0.02 for both), and LVEF showed a trend toward a negative correlation with EAT SFA (*p* = 0.11). As expected, significant correlations were also found between infarct size and LV EDVI (*p* = 0.01), LV ESVI (*p* = 0.0005), and LV EF (*p* = 0.002). In addition, infarct size did not have any significant correlations with EAT FAC. The LV mass index was found to positively correlate with EAT SFA (*p* = 0.04) and negatively correlate with EAT MUFA (*p* = 0.03). These metrics of LV structure and function were not found to significantly correlate with EAT volume or EAT volume index, and likewise were not significantly correlated with the PDFF or FAC of either the VAT or SAT. Furthermore, correlations between EAT FAC measurements and EAT volume and volume index were not found to be significant, although trends were observed.

## 5 DISCUSSION

Whereas prior work has imaged EAT volume and PDFF, to our knowledge the present study represents the first report of FAC MRI of human EAT. Because the EAT composition is altered in conditions such as obesity and plays important roles in inflammation and fibrosis, the quantification of SFA, MUFA, and PUFA by MRI may enable noninvasive detection of proinflammatory EAT. Prior work in a mouse model of obesity showed that treatment with a mineralocorticoid receptor antagonist, a drug with anti‐inflammatory effects in adipose tissue, reduced EAT SFA as measured by FAC MRI and increased perfusion reserve, which supports this hypothesis.^20^ The present findings that EAT FAC metrics in STEMI patients correlate with adverse LV structure and function provide further evidence supporting this hypothesis.

The methods developed in the present study build upon prior work on MRI of fat in and around the heart. Multi‐echo Dixon techniques with two to three TEs have been effective in detecting intramyocardial fat and delineate epicardial and pericardial fat, while allowing for three‐dimensional whole‐heart fat–water imaging.^44^ Accelerated and motion‐corrected extensions of these methods, utilizing low‐rank–based reconstruction techniques, have further facilitated the assessment of cardiac adipose tissue.^45^ The use of three or more echoes, when combined with reconstruction models such as the IDEAL^46^ and VARPRO^47^ methods, allows for multi‐resonance triglyceride models and facilitates the calculation of PDFF in the myocardium and surrounding adipose tissues.^48^, ^49^ Daudé et al.'s recently developed free‐running, IDEAL‐based method enables imaging of PDFF in both the myocardium and nearby adipose depots with radial imaging at 13 TEs, providing improved PDFF measurement accuracy from the larger number of TEs.^50^, ^51^

Whereas FAC MRI methods have been developed previously for other adipose depots, EAT poses unique challenges due to cardiac and respiratory motion and the relatively small size of the depot. Our work addresses these challenges by developing an ECG‐gated sequence to synchronize data acquisition to a consistent portion of the cardiac cycle. To reduce the effects of respiratory motion, the acquisition was shortened to a single breath hold using golden angle radial and local low‐rank acceleration methods. To counter the associated reduction in SNR, a variable flip angle approach was employed to maximize the signal per RF excitation, and the local low‐rank reconstruction was key. The variable flip angle approach was specifically designed for use in adipose tissues and may introduce T_1_ bias associated errors in FAC and PDFF measurements for tissues with significantly different T_1_s. Whereas errors due to T_1_ bias are unlikely to affect adipose tissues with T_1_ values in the range of 250 and 450 ms, tissues containing higher proportions of water may show worse accuracy in PDFF and FAC measurements. Thus, restricting the use of this method to tissues with high PDFF (PDFF >75%), as was done here, is prudent to avoid potential T_1_‐associated measurement error. Replacement of the variable flip angles with a constant, low flip angle would likely mitigate any potential T_1_ effects at the expense of SNR. Further work is needed to understand the detailed effects of T_1_ bias and the range of conditions where it is impactful.

The accuracy of FAC analysis is constrained by voxel size and the quantity of EAT. Larger voxels enhance SNR but risk FAC errors due to partial volume effects from adjacent tissues. Specifically, voxels containing a high water fraction may suffer from the previously discussed effects of T_1_‐associated errors. Voxels with low proton density, such as in the lungs, will have reduced SNR, and voxels with adipose tissue from two separate depots would yield the mean PDFF and FAC of the two depots. Due to these partial volume issues, the most accurate EAT FAC measurements are made in voxels with high PDFF and in central regions of adipose tissue. Cardiac phase further influences the visibility of the EAT, with end‐systole offering optimal EAT visibility and greater separation between the EAT and myocardium. However, the longer period without cardiac motion during end diastole was utilized for data acquisition in the present study because it provided the opportunity for additional RF excitations per heartbeat.

Studies in oil emulsions were employed to validate the FAC MRI method. Whereas MRI FAC estimates generally showed good agreement with reference values, PDFF measurements showed more variablility. Variations in the content of the emulsions that can emerge as they solidify may explain the error in PDFF measurements and the reduction in FAC accuracy for low PDFF emulsions observed during phantom validation.

The measurements made in the present study found distinct FAC profiles for the EAT, VAT, and SAT of STEMI patients. These findings are consistent with known differences in FAC and PDFF between SAT and visceral adipose depots, such as the EAT and VAT, while also highlighting differences in FAC between the EAT and VAT. The FAC phenotype of significantly lower PUFA values in the EAT compared to the SAT aligns with existing nonimaging literature.^40^

The correlations between LV structure and function and EAT FAC in STEMI patients observed in the present study are consistent with recent work that reported a negative correlation between LVEF and EAT volume in similar patients.^41^ Whereas our data did not show the correlation between LVEF and EAT volume, the discrepancy may be explained by the different numbers of patients studied, which were 21 in the present study and 129 in Zhao et al.^41^ Both studies support the hypothesis that EAT plays a role in LV structure and function post‐STEMI. Our data also showed a significant positive correlation between LV mass and SFA, and a significant negative correlation between LV mass and MUFA. As expected, LVEF, LVEDVI, and LVESVI were strongly correlated with infarct size; however, EAT FAC and infarct size showed no significant correlations. These results potentially indicate that, in the studied population, LV structure and function are associated with EAT FAC in a way that is independent of infarct size.

Whereas existing abdominal FAC methods provide valuable insights into overall adiposity and visceral adipose tissue composition, the value of EAT‐specific measurements lies in the localized assessment of a region with direct proximity to the myocardium. Prior work has shown the use of imaging EAT volume or thickness for patient outcome prediction and risk stratification—and also for understanding the role of EAT in atrial fibrillation, coronary microvascular disease, and coronary artery disease.^5^, ^6^, ^52^, ^53^ Beyond EAT quantity, there is also interest in imaging EAT quality. For example, utilization of EAT radiodensity by cardiac CT has emerged as a valuable parameter for such applications.^54^, ^55^, ^56^, ^57^ Notably, Franssens et al. observed that EAT radiodensity is associated with an adverse cardiovascular risk factor profile in patients at high risk of cardiovascular disease, independent of the volume of epicardial adipose tissue.^57^ Like EAT radiodensity, EAT FAC and PDFF may also potentially serve as better prognostic and modifiable biomarkers than EAT volume. Investigation of EAT PDFF is at a nascent stage and holds potential as a biomarker of hypertrophied adipocytes and proinflammatory EAT. Furthermore, there are known biological functions linking SFAs to proinflammatory signaling,^12^ and EAT SFA levels have been shown to be modifiable through pharmacological therapy.^20^ This suggests that the measurement of EAT SFA levels could potentially serve as a clinically important biomarker for proinflammatory EAT.

The proposed FAC MRI method has limitations, notably related to signal model simplifications that impact the accuracy of FAC MRI. The determination of the field inhomogeneity map involves making assumptions about phase smoothness, which could result in inaccuracies at interfaces such as the lung‐EAT boundary. Additionally, flow artifacts from blood vessels might contribute to local phase discrepancies. Because the accuracy of FAC MRI relies on the accuracy of the field map, these potential sources of error could affect the precision of FAC measurements in specific regions. Whereas the use of a bounded solution to the signal model via constraint of the ndb to nmidb ratio improves the robustness of the method, it limits its ability to be sensitive to large MUFA‐ or PUFA‐specific changes in the FAC that could potentially exist outside of the constrained ranges. Further improvements to the proposed FAC MRI method that improve accuracy and reproducibility and increase spatial coverage of the heart are key areas for further development. Employing techniques such as free‐breathing and/or free‐running sequence designs and image reconstruction processes may allow for higher SNR and for images to be collected at additional TEs, slice positions, and cardiac phases. More slices would increase the spatial coverage, and more TEs could potentially reduce the number of model assumptions needed for accurate FAC estimation.

## 6 CONCLUSION

A novel FAC MRI approach for characterizing EAT provides insights into its fatty acid composition and its potential role in assessing obesity‐related metabolic cardiovascular diseases. The developed method addresses challenges specific to EAT imaging, including cardiac and respiratory motion, and demonstrates promising results in phantom validation and in vivo studies. The observed associations between LV function and structure and EAT FAC in STEMI patients could signify the potential of these methods to discern variations in EAT composition that may be diagnostic or prognostic. Whereas the method has limitations, ongoing refinements and advancements in imaging technology may enhance its accuracy and broaden its applications in understanding the role of EAT in cardiovascular disease.

## FUNDING INFORMATION

Supported by the National Heart, Lung, and Blood Institute (NHLBI), grant R01 HL162872; the National Institute on Aging (NIA), grant R01 AG076360; the American Heart Association (AHA), grant 23PRE1011202; and the National Institutes of Health (NIH), grant T32 HL007284.
